# Steroid treatment response combined with serological mark in differentiating type-1 autoimmune pancreatitis from pancreatic cancer

**DOI:** 10.1097/MD.0000000000031660

**Published:** 2022-11-11

**Authors:** Bingqian Liu, Ning Tang, Yuan Yao, Hua Li, Lishan Xu, Bin Zhou, Bin Liu

**Affiliations:** a Department of Rheumatology, The Affiliated Hospital of Qingdao University, Qingdao, Shandong Province, China; b Department of Nutrition, Weifang People’s Hospital, Shandong Province, China; c Department of Biliary and Pancreatic Surgery, Department of Retroperitoneal Tumor Surgery, The Affiliated Hospital of Qingdao University, Qingdao, Shandong Province, China.

**Keywords:** differential diagnosis, pancreatic cancer, serology, treatment, type-1 autoimmune pancreatitis

## Abstract

Autoimmune pancreatitis (AIP) and pancreatic cancer (PC) are two different diseases. Their diagnosis, treatment and prognosis are different, and it is difficult to differentiate them. This study aimed to explore the role of steroid treatment response combined with serological mark in distinguishing type-1 AIP from PC. Clinical data were collected and compared from 50 cases of AIP (group 1) and 100 cases of PC (group 2). The diagnostic value of serum IgG4, CA19-9, globulin (GLB) and eosinophil cell (EC) were evaluated. The response of steroid treatment of 28 patients with atypical imaging in group 1 was analyzed. After 2 weeks, the patients were classified as positive and negative steroid response according to the manifestations and/or the radiological changes. The positive response cases (n = 20) were confirmed as AIP, whereas negative ones (n = 8) were finally diagnosed as PC after complete resection. Serum GLB, IgG4 and EC levels in group 1 were significantly higher than those in group 2 (P < .01), and CA19-9 levels were distinctly lower in group 1 (P < .01). The level of serum IgG4 was related to the accuracy of diagnosis of AIP on the basis of the result of logistic regression analysis. Two-weeks steroid therapy response combined with serum IgG4 levels contribute to the differential diagnosis AIP and PC. However, regular and long-term follow-up were importance for the differential diagnosis. There was an urgent need to explore the specific markers that distinguish these 2 entities.

## 1. Introduction

Autoimmune pancreatitis (AIP) and pancreatic cancer (PC) were 2 diverse entities, which occurred in older men with similar clinical manifestations such as obstructive jaundice.^[1–3]^ AIP was related to autoimmunity that responded dramatically to steroid therapy, whereas PC was generally surgical resected^[4]^ with a 5% mortality and 40% to 50% morbidity.^[5]^ This differentiation was critical as the management and prognosis of the 2 diseases vary drastically.

AIP was a rare type of chronic pancreatitis, which was divided into type 1 and type 2 on the basis of pathological manifestations.^[6]^ Type-1 AIP was considered as a part of systemic IgG4-related disease that could involve extra-pancreatic organs accounting for 96% of Asian AIP cases.^[6,7]^ Therefore, the AIP discussed in our study refers to type 1. Besides, PC was a malignant tumor with a 5-years survival rate of only about 7%.^[8]^ It was usually asymptomatic in the early stages, only about 20% to 30% of PC patients benefited from potentially curative surgical resection.^[4]^ Although some advance has been made and several diagnostic criteria have been proposed for AIP, the differential diagnosis from PC is still a great challenge in clinical practice, especially when the imaging features are atypical. Unfortunately, 2.5% to 11% of AIP patients were undergo unnecessary surgery for presumed PC.^[9,10]^

According to the 2011 International Consensus Diagnostic Criteria (ICDC), for AIP patients with atypical imaging findings, endoscopic pancreatogram, other organ involvement (OOI), serum IgG4 levels, histology of the pancreas and response to steroids could be used to clarify the diagnosis.^[11]^ The ICDC criteria were proposed to use serum IgG4 alone as the serological criterion for AIP, which was usually elevated.^[12]^ However, a significant minority of AIP cases might have normal serum IgG4 and 7% to 10% of PC cases exhibited false-positive elevation.^[13]^ In addition, since the lower prevalence of AIP, the positive predictive value of IgG4 for the diagnosis of AIP was estimated to be only 80%, which might limit its application.^[14]^ Therefore, recent studies have been devoted to explore the usefulness of IgG4 combined with other serological parameters.^[13,15,16]^ To date, few researches had been conducted to evaluate the utility of diagnostic steroid treatment response for distinguishing these 2 entities.^[17]^ We aimed to verify the validity of steroid treatment response combined with serological marks so that summarize an effective and convenient strategy of differential diagnosis.

## 2. Materials and methods

### 2.1. Preliminary screening to exclude malignant disease before enrollment

Before diagnostic steroid treatment, preliminary screening was performed to exclude malignant disease. Abdominal computed tomography (CT) and magnetic resonance cholangiopancreatography (MRCP) even PET-CT was carried out. Serum tests such as IgG4, CA19-9 and other tumor markers were also performed. Only patients with negative results for malignant diseases were enrolled in steroid treatment.

### 2.2. Study subjects

The subjects of combined serological test were 50 cases of AIP (group 1, including 22 of typical imaging and 28 of atypical imaging) and 100 cases of PC (group 2) who visited the Affiliated Hospital of Qingdao University in China from December 2012 to December 2019. The diagnosis of AIP was determined according to the 2011 ICDC.^[11]^ PC patients were selected to match in age and sex.

After the initial assessment, 22 patients (20 men and 2 women) had typical imaging for AIP, which was defined as diffuse enlargement with delayed enhancement and long or multiple strictures of the main pancreatic duct without marked upstream dilatation.^[11]^ They were treated with steroids adjusted by the condition. Prednisolone was reduced by 5 to 10 mg/d every 1 to 2 weeks until the daily dosage was 20mg, followed by tapering with 5 mg every 2 weeks. A dose of 5 to 10 mg/d was maintained for over 2 years before steroid could be discontinued. The subjects of diagnostic steroid treatment were the remaining 28 patients with atypical imaging, which were prospectively managed by means of oral prednisolone 0.6 to 1.0 mg/kg per day. Abdominal CT, MRCP, IgG4 and CA19-9 were reviewed 2 weeks later to assess steroid responsiveness. The study was approved by the institutional review board of the Affiliated Hospital of Qingdao University. Informed consent was obtained from every patient.

Positive steroid response was defined as complete resolution or noticeable improvement of the main pancreatic duct stenosis and the pancreatic mass in imaging, as well as reduced CA19-9 levels. On the contrary, negative steroid response was regarded as no improvement in the main pancreatic duct stenosis or pancreatic mass, and increased CA19-9 level. In the patients of positive response, steroid therapy was continued if they were confirmed as AIP combined with OOI, serum IgG4 and/or histology. As for the cases presenting with negative response, steroid management was discontinued and surgical exploration was performed subsequently. A final diagnosis was obtained by surgical exploration or long-term follow-up.

### 2.3. Serological parameters

All of the serum parameters were measured simultaneously as the routinely performed test on the first visit including hemoglobin (Hb), eosinophil cell (EC), globulin (GLB), total bilirubin, direct bilirubin, alanine aminotransferase, aspartate aminotransferase, alkaline phosphatase, *γ*-glutamyltransferase, fasting blood glucose (FBG), IgG4, and CA19-9.

### 2.4. Statistical analysis

We used IBM SPSS Statistics 20.0 for the statistical analysis. Kolmogorov-Smirnov test was used to detect whether the quantitative data was normally distributed. Continuous variables were expressed as a median value of interquartile range. For comparisons of qualitative data the chi-squared test was adopted, while the Student *t*-test or Mann–Whitney *U*-test were applied to comparisons of quantitative variables. Receiver operating characteristic (ROC) analysis was carried out to assess the diagnostic value of serum parameters and their optimal cutoff levels. Logistic regression analysis was conducted to calculate the simultaneous effect of the serum parameters. The *P*-value < 0.05 (2-tailed) was considered statistically significant.

## 3. Results

In our study, all patients with suspected diagnosis of AIP underwent serum tumor marker examinations and chest + abdominal CT examinations to exclude malignant diseases before treatment, and 5 of them completed PET-CT examinations. More than half of the patients had 2-flod increment above upper normal limit serum IgG4 subclass levels.

In terms of OOI, there were 3 cases of submandibular gland involvement, 1 case of hilar + mediastinal lymph node involvement, 1 case of lacrimal gland + submandibular gland + extraocular muscles involvement, 1 case of parotid gland + submandibular gland involvement, 1 case of extraocular muscle involvement, and 1 case of parotid gland involvement; 1 case of gallbladder + abdominal aorta involvement, and AIP-related biliary changes were found in 9 cases.

Regarding the pathological examinations, a total of 5 patients accepted histological examinations, including 2 cases of intraoperative pathology, 1 case of pancreatic lump biopsy, 1 case of left orbital biopsy, and 1 case of right submandibular gland biopsy. Among them, 3 patients were diagnosed as AIP after supplementary immunohistochemical examination.

Basic characteristics and serological parameters of AIP and PC were shown in Table 1. Forty-five males and 5 females were included in group 1, while 71 males and 29 females were in group 2. Both AIP and PC were more common in older men, while the age of PC patients was a little older than AIP patients (*P* < .05).

**Table 1:** Characteristics of subjects and serological parameters.

Variable	AIP (n = 50)	PC (n = 100)	*P* value
Age (yr)	60 (49–65.25)	62 (54–69)	.034
Male (N%)	45 (90%)	74 (74%)	.102
CA 19-9 (U/mL)	46.87 (23.40–77.15)	165.40 (41.71–441.73)	<.001
EC (10^9^/L)	0.21 (0.09–0.57)	0.11 (0.05–0.17)	<.001
Hb (g/L)	125.50 (120–138)	134 (121–143)	.158
GLB (g/L)	31.90 (27.21–36.63)	25.60 (21.60–28.36)	<.001
FBG (mmol/L)	6.43 (5.04–9.69)	5.80 (5.13–7.16)	.362
IgG4 (g/L)	3.94 (3.59–4.75)	0.55 (0.31–1.11)	<.001

Age and laboratory tests in median (interquartile range).

AIP = autoimmune pancreatitis, EC = eosinophil cell, FBG = fasting blood glucose, GLB = globulin, PC = pancreatic cancer.

**Figure 1.**
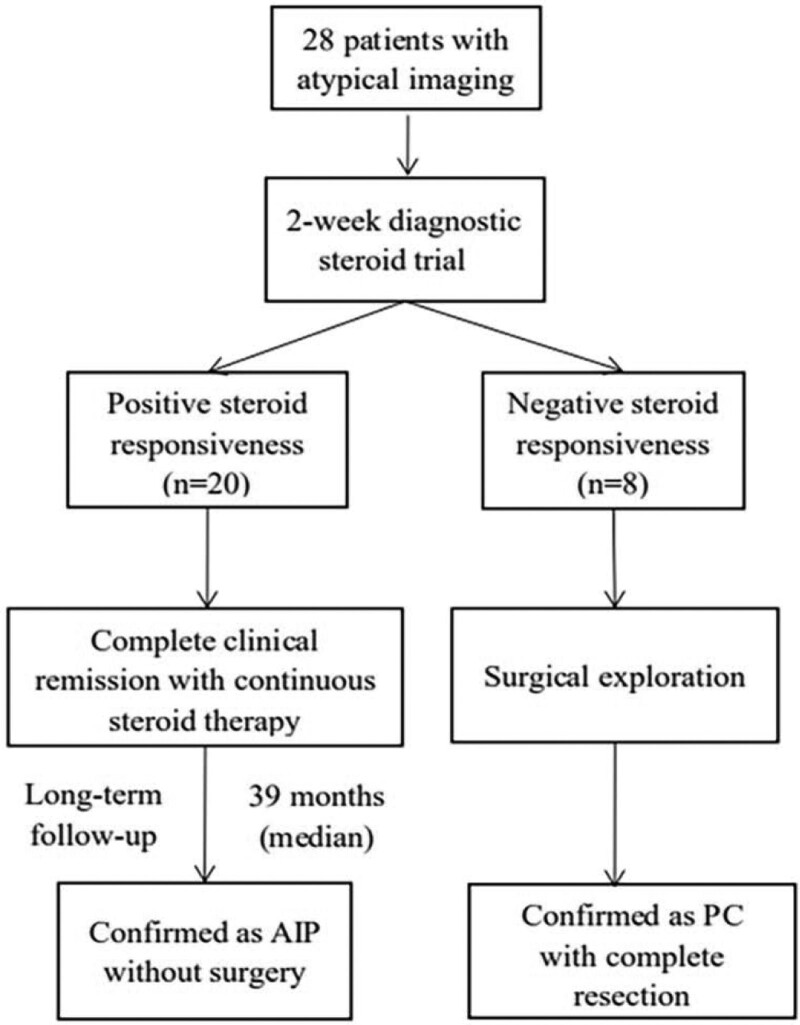
The study flow chart of steroid diagnostic therapy.

**Figure 2.**
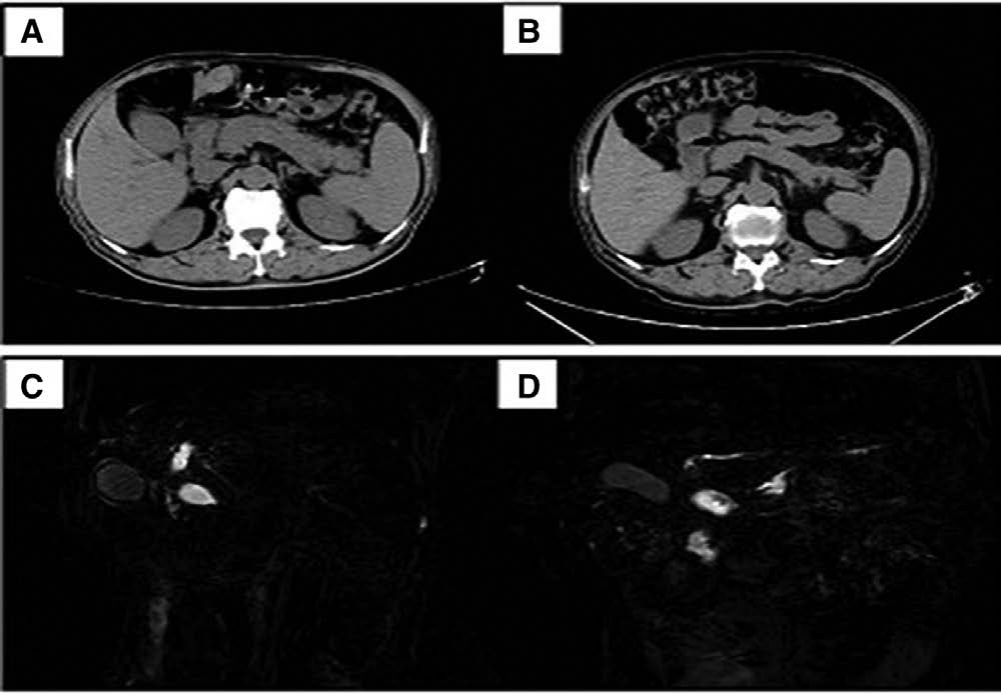
Serial images from a 67-years old man with positive steroid responsiveness who was finally diagnosed as type-1 autoimmune pancreatitis. (A, C) Pretreatment: CT showed diffuse enlargement of the pancreas, and endoscopic MRCP showed narrowing of the main pancreatic duct. (B, D) Post-treatment: after the 2-weeks steroid trial, the pancreas was reduced in size and the main pancreatic duct stenosis was significantly improved. CT = computed tomography, MRCP = magnetic resonance cholangiopancreatography.

With the 2-weeks steroid treatment, 20 (18 men and 2 women) of 28 patients presented a positive response to steroids. It took 1 to 2 months for patients to achieve complete remission, which was estimated by clinical manifestation, imaging and serological changes. During a median follow-up of 39 months (range 6–63 months), the 20 patients were ultimately confirmed as AIP and none of the patients developed malignant tumors. The results of diagnostic steroid treatment were integrated in Figure 1. The imaging of an AIP patient with positive steroid response were showed in Figure 2. The rest 8 patients were regarded as negative steroid response, pancreatic surgery was performed on immediately. Final diagnosis of PC was made in these patients on the basis of histopathology. During a meanone year follow-up, 6 patients of them were still alive.

The statistical results of routine laboratory tests such as complete blood count and biochemical tests showed significant differences in EC, GLB, IgG4 and CA19-9 between the 2 groups. EC, GLB and IgG4 levels were prominently higher in group 1 than that in group 2 (*P < *.001). In contrast, CA19-9 levels were significantly lower in group 1 than that in group 2 (*P *< .001). However, the levels of alanine aminotransferase, aspartate aminotransferase, alkaline phosphatase, *γ*-glutamyltransferase, total bilirubin, direct bilirubin, FBG and Hb had no remarkable distinction between the 2 groups.

**Figure 3.**
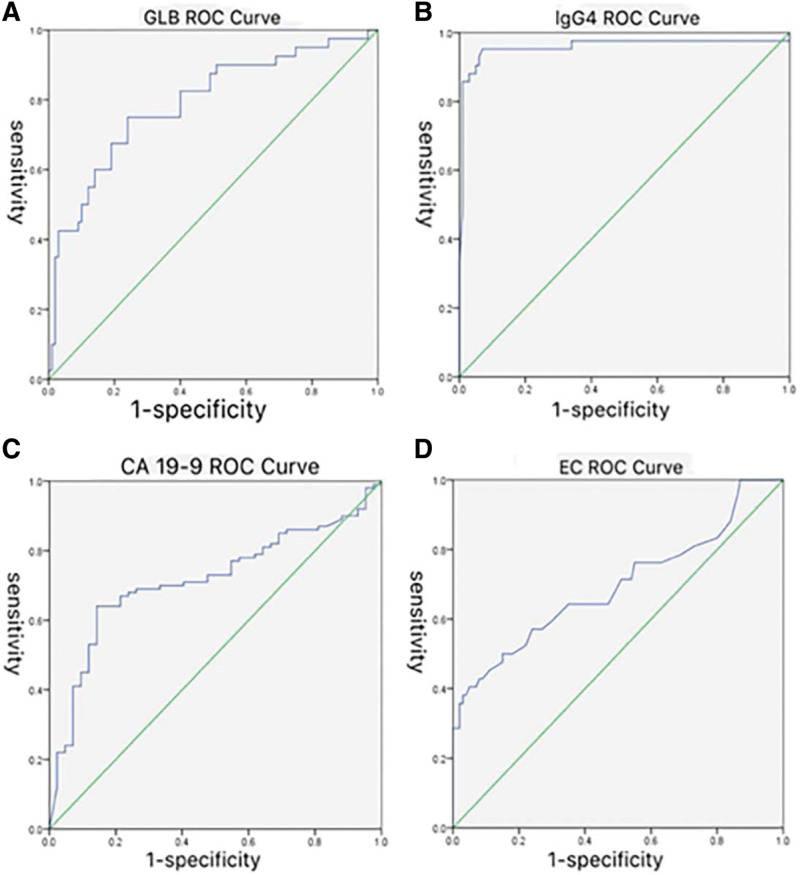
Receiver operating curve of GLB, IgG4, CA19-9 and EC. EC = Eosinophil cell, GLB = Globulin.

ROC curves were conducted to differentiate AIP from PC based on the levels of EC, GLB, IgG4 and CA19-9 (Fig. 3A–D). As shown in Table 2, the optimal concentration for EC was determined as 0.30 × 10^9^/L, the sensitivity was 40.5%, and the specificity was 99.5%, while AUC was 0.688. The area under the curve (AUC) for GLB was 0.791 at an optimal concentration of 28.18 g/L with the sensitivity 75.0% and the specificity 76.0%. IgG4 at the cutoff concentration of 1.96 g/l showed the sensitivity of 95.2% and specificity of 99.3%, with AUC reaching 0.982. The sensitivity and specificity of CA19-9 were 64% and 86%, respectively at the optimal concentration of 90.09 U/mL, with AUC of 0.715. Logistic regression analysis was applied to verify the comprehensive diagnostic value of these serological markers. The result revealed that only the level of IgG4 was related to the diagnosis of AIP, which was listed in Table 3.

**Table 2:** Diagnostic characteristic of serological parameters to differentiate AIP and PC.

Variable	AUC (95% CI)	Sensitivity	Specificity	*P* value
GLB > 28.18 g/L	0.791 (0.703–0.878)	75%	76%	<.0001
IgG4 > 1.96 g/L	0.982 (0.961–1.000)	95.2%	99.3%	<.0001
CA19-9 < 90.09 U/mL	0.715 (0.628–0.802)	64%	86%	<.0001
EC > 0.30 × 10^9^/L	0.688 (0.579–0.797)	40.5%	99.5%	.001

AIP = autoimmune pancreatitis, EC = eosinophil cell, GLB = globulin, PC = pancreatic cancer.

**Table 3:** Results of the logistic analysis for AIP.

Variable	*β*	SE	Wald value	*P* value
IgG4	–2.355	0.439	28.732	<.0001
Constant	6.020	1.068	31.757	<.0001

*β* = partial regression coefficient; AIP = autoimmune pancreatitis, SE = standard of partial regression coefficient.

## 4. Discussion

To different AIP from PC was still a hard task in clinical practice, due to similar clinical manifestations of 2 entities. The AIP patients with typical imaging were being diagnosed with increasing frequency attributed to increasing awareness.^[18]^ However, it was still difficult to distinguish focal massforming AIP from PC which accounted for 28% to 41% of cases of AIP.^[19]^ Once misdiagnosed, patients would suffer unnecessary trauma and pain.

For AIP patients manifesting as a focal mass or focal enlargement, histopathological feature was generally regarded as the gold standard. Because of the small size of pancreatic tissue obtained through fine needle aspiration, endoscopic ultrasonography was difficult to diagnose AIP. Central biopsies, though efficient in obtaining adequate pancreatic tissue, had not been widely performed^.[20,21]^ ERCP was applied to assess the cholangiopancreatic system. However, many endoscopists avoided injecting the pancreatic duct in patients with obstructive jaundice afraid of causing pancreatitis.^[11]^ MRCP was insensitive to the differences between AIP and PC. Although PET-CT scans could be used for early diagnosis and differential diagnosis of tumors, it could not distinguish AIP from early PC.

Diagnostic treatment and serological tests could assist in helping distinguish the entities. Though response to therapy was adopted as a collateral evidence in several criterion, few study had been designed to testify its utility till now. In our study, steroid was only given to clinically suspected AIP patients with atypical imaging after initial investigation for malignant disease. Among the 28 patients, 20 cases (71%) had positive response to steroid and were finally diagnosed as AIP. 8 cases negative response to steroid (29%) were confirmed as PC based on pathology. Similar to our study, the study of S-H Moon et al showed that 15 of 22 patients (68%) with positive steroid response obtained the diagnosis of AIP and 7 patients (32%) of PC manifested negative response to steroid.^[17]^

Although AIP was sensitive to steroid therapy, the application of steroids was usually premised on the exclusion of malignant disease, which limited the usefulness of diagnostic steroid treatment. Radiological improvement of AIP might occur as early as 1 to 2 weeks after steroid therapy.^[22–24]^ Therefore, the effectiveness of steroid for patients with AIP could be reflected within 2 weeks. Given PC was so highly malignant tumor that the overall survival rate was 28% after 1 year and 7% after 5 years. Moreover, the resection rate was only 20%, a 2-weeks observation might not have an adverse impact on the surgical outcome of potentially resectable PC. Besides, Benz et al showed that steroid could inhibit the proliferation of pancreatic exocrine cells of rats, thus delaying the progression of PC.^[25]^ Norman et al also suggested that steroid could restrain the proliferation of human PC cells.^[26]^ Then we encouraged 2-weeks steroid treatment, patients of positive steroid response were eventually diagnosed with AIP, while the patients with PC showed negative steroid response. Thus, the effectiveness of the steroid trial in differentiating the 2 diseases could be found in our observation.

Serum IgG4 level was a meaningful diagnostic parameter that could be elevated in cases of AIP, with 67% to 95% of sensitivity and 89% to 100% of specificity.^[27–29]^ In our research, compared to group 2, IgG4 levels in group 1 were significantly increased. The sensitivity and specificity of IgG4 were 95.2% and 99.3% respectively, consistent with other studies. However, 10% of patients with PC had elevated IgG4 levels (>140 mg/dL), and in 1% to 2.4% of patients, IgG4 levels were twice as high as the normal upper limit. Therefore, serum IgG4 concentration alone lacked adequate value for differential diagnosis of AIP and PC.^[12,30]^

Serum CA19-9 level was the most useful marker for PC with a sensitivity and specificity of 79% and 82% separately,^[31]^ and more often elevated in PC than in AIP patients.^[32]^ Our study revealed that levels of CA19-9 increased more significantly in PC. However, it could also be positive in other benign diseases except for AIP.^[6]^ CA19-9 was also less sensitive to PC with a smaller diameter (≤2 cm), which was the biggest diagnostic challenge in distinguishing PC from focal AIP. It’s worth noting that the optimal cutoff value of CA19-9 was much higher than used in the clinical practice for PC screen. Compared to previous studies, the optimal concentration was 85 U/mL^[15]^ and 306.75 U/mL^[33]^ respectively, which showed that higher cutoff concentration might increase the diagnostic accuracy.

EC have also been described in IgG4-related disease.^[34]^ A clinical history of allergy was ascertained in 40 to 80% of AIP.^[35]^ Our study proved that EC in group 1 was indeed higher. But the sensitivity was too lower to be a diagnostic marker singly. Prior studies had shown that patients with both AIP and PC had mild anemia, and AIP patients had lower hemoglobin levels.^[36]^ However, in our study, there was no remarkable difference between the 2 groups, which might be related to the severity of illness and the number of AIP patients. Ayush Sharma et al discovered that FBG level was associated with PC diagnosis time, tumor volume and grade.^[37]^ Patients had an average of 36 to 30 months of hyperglycemia before the diagnosis of PC, suggesting its underlying differential diagnostic value. We measured FBG levels of AIP and PC patients at the first visit, however the results showed no distinct difference between the 2 groups.

To verify the diagnostic value of serological parameters, ROC analysis was performed in all AIP and PC patients. It was obvious from the results that any single serum marker was not enough for diagnosis. Logistic regression analysis was used to explore the simultaneous effects of EC, GLB, IgG4, and CA19-9. It was observed that only IgG4 had the diagnostic value for AIP, the other 3 parameters, though different between the 2 diseases, were not associated with differential diagnosis. Specific markers used to differentiate the 2 entities were in urgent need to be explored.

## 5. Conclusion

In the clinical suspected AIP patients with atypical imaging finding, 2-weeks diagnostic steroid therapy and serum IgG4 could help differentiate it from PC. However, regular review and long-term follow-up were indispensable. There was an urgent need to explore the specific markers that distinguish these 2 entities.

## Author contributions

**Conceptualization:** Bingqian Liu, Ning Tang.

**Data curation:** Lishan Xu.

**Formal analysis:** Yuan Yao, Bin Zhou.

**Investigation:** Yuan Yao, Hua Li.

**Software:** Lishan Xu.

**Supervision:** Yuan Yao, Hua Li, Bin Zhou.

**Writing – original draft:** Bingqian Liu, Ning Tang.

**Writing – review & editing:** Bin Zhou, Bin Liu.
